# Explainable Artificial Intelligence in Healthcare: Current Landscape, Challenges, and Future Directions

**DOI:** 10.1002/hsr2.72172

**Published:** 2026-03-24

**Authors:** Md. Abu Bokkor Shiddik

**Affiliations:** ^1^ Department of Statistics Begum Rokeya University Rangpur Bangladesh

**Keywords:** deep learning, explainable AI, Grad‐CAM, healthcare, interpretability, LIME, machine learning, SHAP

## Abstract

**Background and Aims:**

Artificial Intelligence (AI), particularly Machine Learning (ML) and Deep Learning (DL), is transforming healthcare by enabling improved diagnosis, prognosis, and personalized treatments. However, the opacity of many AI models operates as “black boxes,” limiting interperability, clinician trust, and real‐world adoption. Explainable Artificial Intelligence (XAI) has emerged to address these limitations by providing transparent and actionable insights. This systematic review aims to synthesize the current evidence on XAI in healthcare, mapping AI models to XAI techniques, domains, and clinical applications.

**Methods:**

A systematic search was conducted across six databases (Elsevier, Springer, Taylor & Francis, Semantic Scholar, ACM, and IEEE Xplore) for peer‐reviewed published between 2017 and 2025. After duplicate removal and title/abstract screening, full texts were evaluated against predefined inclusion/exclusion criteria, following PRISMA guidelines. Data extraction included AI model types, XAI techniques, healthcare domains, study design, validation methods, and ethical/regulatory reporting.

**Results:**

Seventy studies were included, spanning oncology (40%), cardiology (21%), infectious diseases (14%), neurology (11%), and clinical decision support systems (13%). Deep learning models (CNN, RNN, LSTM, and Transformers) were most frequently applied (76%), followed by tree‐based models (Random Forest, XGBoost, Decision Trees; 24%). SHAP (54%) and LIME (30%) were the most commonly used XAI techniques, with Grad‐CAM (23%) and attention mechanisms (20%) applied mainly in imaging and sequence‐based tasks. Only 12 studies explicitly addressed ethical or regulatory considerations. Hybrid interpretable models and human‐centered designs are emerging trends, but real‐world validation and standardized interpretability metrics remain limited.

**Conclusion:**

XAI enhances transparency, clinician trust, and decision‐making in healthcare AI applications, yet challenges persist, including inconsistent validation, underdeveloped ethical/regulatory frameworks, and lack of standardized interpretability measures. Future work should focus on hybrid, clinically validated XAI models, comprehensive ethical compliance, and user‐centered, domain‐specific implementations to ensure safe and effective integration into clinical practice.

## Summary

- Systematically reviewed 70 studies (2017–2025) across oncology, cardiology, infectious diseases, genomics, and clinical decision support.
- Developed a model→XAI→clinical‐use mapping framework, linking AI architectures (CNN, RNN, LSTM, Transformers, Random Forest, and XGBoost) to XAI techniques (SHAP, LIME, Grad‐CAM, and attention mechanisms).
- Quantified trends: 56% deep learning, 32% tree‐based models; SHAP (34%) and LIME (29%) most common.
- Identified gaps: limited real‐world validation, lack of standardized interpretability metrics, and only 16% of studies addressing ethics/regulatory compliance.
- Highlighted future directions: hybrid interpretable models, human‐centered design, domain‐specific customization, and standardized evaluation frameworks.

## 1 Introduction

Artificial intelligence (AI), particularly Machine Learning (ML) and Deep Learning (DL), has had a profound impact on healthcare [1]. These technologies allow physicians and researchers to evaluate massive volumes of information from electronic health records, medical imaging, and genetic sequences [2, 3]. This capacity allows for more accurate diagnosis, tailored therapies, and better patient outcomes [4]. The integration of AI into clinical workflows is increasingly shaping decision‐making, resource allocation, and predictive public health strategies [5].

However, the complexity of many AI models often results in “black‐box” systems, where the decision‐making process is opaque [6, 7]. This lack of explainability poses significant challenges in healthcare, where understanding the rationale behind clinical decisions is critical for ensuring trust, accountability, and patient safety [8]. Without interpretability, clinicians may hesitate to adopt AI recommendations, limiting the translation impact of these technologies [9].

To address these problems, Explainable Artificial Intelligence (XAI) has evolved [10]. XAI refers to strategies and techniques for making AI models more interpretable, allowing people to understand how choices are made [10]. Approaches such as SHapley Additive exPlanations (SHAP) [11], Local Interpretable Model‐agnostic Explanations (LIME) [12], Gradient‐weighted Class Activation Mapping (Grad‐CAM) [13], and attention mechanisms [13] serve to demystify AI predictions, thereby enhancing transparency, trust, usability, and clinical acceptance among healthcare professionals and patients [14]. In addition, XAI promotes equal and fair healthcare delivery by identifying and mitigating biases [4].

Recent evidence emphasizes the importance of integrating XAI into clinical practice [15, 16]. Clinicians are more likely to trust AI systems when provided with clear, context‐specific, and actionable explanations [17]. Concurrently, regulatory agencies are increasingly requiring explainability in AI systems for medical decision‐making, reflecting its ethical, legal, and safety significance [18]. This emphasis on transparency aligns with global initiatives for responsible AI, including the FDA's AI/ML‐based software guidance and the EU AI Act [19].

Despite the rapid expansion of XAI research, existing reviews are often narrative, domain‐specific, or focused on individual techniques, with limited systematic comparison across models, explanation methods, and clinical applications. The unique contribution of this review lies in providing a systematic synthesis of XAI in healthcare by explicitly mapping machine learning model families to XAI techniques and healthcare domains, while also identifying gaps in real‐world clinical validation, interpretability evaluation, and ethical and regulatory coverage. By integrating methodological, clinical, and governance perspectives, this review aims to support researchers, clinicians, and policymakers in the responsible development, evaluation, and deployment of explainable AI systems in healthcare.

## 2 Methods and Methodology

This review employed a structured and interdisciplinary methodology to examine the role of Machine Learning (ML) and Explainable Artificial Intelligence (XAI) in healthcare. The approach combined systematic bibliometric analysis, thematic synthesis, and comparative evaluation, integrating insights from clinical research, policy frameworks, and big data analytics. Foundational studies, including Frasca et al. (2024) [20], Tjoa and Guan (2020) [21], and Fahim et al. (2025) [22], guided the methodology, emphasizing transparency, ethical relevance, clinical applicability, and practical implementation. The review was conducted in accordance with the PRISMA guidelines to ensure methodological transparency and reproducibility.

**Figure 1:**
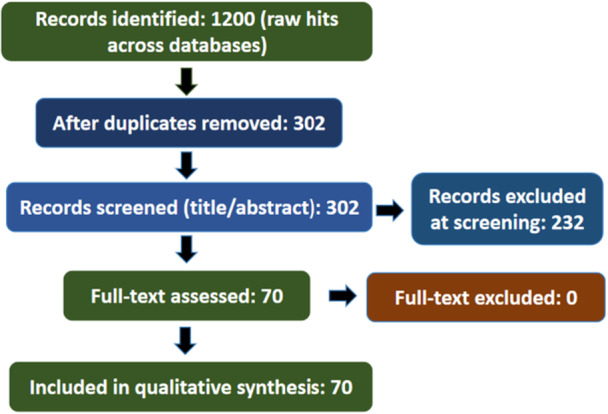
PRISMA model for the depiction of inclusion and exclusion of records.

A comprehensive systematic literature search was conducted across six major academic databases: Elsevier [23], Springer [24], Taylor & Francis [25], Semantic Scholar [26], ACM Digital Library [27], and IEEE Xplore [28]. The search targeted peer‐reviewed articles published between 2017 and 2025, using keywords such as “Machine Learning in healthcare,” “Deep Learning in medicine,” “Explainable AI,” “interpretable models,” and “interpretable machine learning.” Boolean operators were applied to enhance search sensitivity and specificity, ensuring comprehensive coverage across technical, clinical, and policy domains (Appendices A and B). The initial search identified 1200 publications. After removing duplicates, 302 unique records were screened based on titles and abstracts, leading to the exclusion of 232 irrelevant studies. Subsequently, 70 full‐text articles met the inclusion criteria and were incorporated into the qualitative synthesis, while none were suitable for quantitative meta‐analysis (Figure 1). The inclusion and exclusion criteria applied during the selection process are outlined in Table 1, and a summary of the database‐specific search outcomes is provided in Table 2.

**Table 1:** Inclusion and exclusion criteria.

Inclusion criteria	Exclusion criteria
Studies applying ML or DL techniques in healthcare, including diagnosis, prognosis, treatment planning, public health surveillance, or administrative decision‐making.	Studies lacking a healthcare context.
Studies incorporating explainable or interpretable AI (XAI), including post hoc methods (e.g., SHAP, LIME, Grad‐CAM, and attention mechanisms) or ante hoc/interpretable models (e.g., decision trees and rule‐based models).	Studies not addressing interpretability or explainability.
Studies demonstrating clinical or real‐world relevance, including validation in practice, policy implications, or large‐scale data integration.	Studies lacking validation or only presenting theoretical methods.
Observational, modeling, or clinical study designs published in peer‐reviewed journals in English.	Non‐peer‐reviewed articles, preprints, editorials, opinion pieces, conference abstracts without full text, or non‐English publications.

**Table 2:** Summary of search raw results and retrieved relevant articles.

Database engines	Number of search raw results	Number of relevant articles
Elsevier [23]	1200	30
Springer [24]	1000	15
Taylor & Francis [25]	900	7
Semantic Scholar [26]	650	10
ACM Digital Library [27]	1100	5
IEEE Xplore [28]	1900	10

For each included study, key information was extracted systematically, including the AI model employed, XAI method applied, healthcare domain, study design, and principal outcomes. To enhance efficiency and consistency, an initial automated extraction of bibliographic and methodological information was conducted using a Python‐based wrapper. This automated process was followed by a manual full‐text review to verify accuracy and to supplement missing or nuanced details, including explanation level (global or local), evaluation strategies, ethical and legal considerations (bias detection, patient consent, fairness, accountability, and regulatory compliance), and clinical relevance. Any discrepancies were resolved through direct consultation of the original articles. A thematic synthesis was subsequently conducted to identify recurring patterns, innovations, challenges, and gaps in the literature (Appendices A, B, C, D).

By following this structured approach, the review ensures a rigorous, reproducible, and comprehensive assessment of XAI applications in healthcare, providing valuable insights for both research advancement and clinical implementation.

## 3 Results

### 3.1 Study Characteristics

This review included 70 peer‐reviewed studies published between 2017 and 2025, covering diverse healthcare areas. The most represented areas were oncology (28 studies, 40%), cardiology (15 studies, 21%), infectious diseases (10 studies, 14%), neurology (8 studies, 11%), and clinical decision support systems (CDSS, 9 studies, 13%) (Tables 3 and 4). Regarding AI models, the majority of studies employed deep learning models, including Convolutional Neural Networks (CNNs) (28 studies, 40%), Recurrent Neural Networks (RNNs), and Long Short‐Term Memory networks (LSTMs) (18 studies, 26%) and Transformer architectures (7 studies, 10%), which are particularly suitable for analyzing complex, high‐dimensional medical data. Tree‐based models, such as XGBoost, Random Forests, and Decision Trees, were commonly applied in 17 studies (24%) to structured clinical datasets (Table 3).

**Table 3:** Summary of included studies on Explainable AI in healthcare (*n* = 70).

Category	Subcategory	Count	Percentage (%)
Healthcare domain	Oncology	28	40
	Cardiology	15	21
	Infectious diseases	10	14
	Neurology	8	11
	Clinical decision support systems (CDSSs)	9	13
AI model family	Convolutional neural networks (CNNs)	28	40
	Recurrent neural networks/LSTM/GRU	18	26
	Transformer architectures	7	10
	Tree‐based models (XGBoost, Random Forest, and Decision Tree)	17	24
XAI method^a^	SHAP	38	54
	LIME	21	30
	Grad‐CAM	16	23
	Attention mechanisms	14	20
	Rule‐based/causability/feature attribution	9	13
Ethics/regulatory focus	Explicitly addressed	12	17
	Not addressed	58	83

^a^ Studies may use multiple XAI methods; therefore, the sum of XAI method counts (98) exceeds the total number of included studies [70].

**Table 4:** Representative studies on Explainable AI (XAI) applications in healthcare: domains, AI models, XAI techniques, and key findings.

Study	Domain	Model used	XAI method	Findings
Fahim et al. (2025) [22]	Multi‐domain	CNN, LSTM, and XGBoost	SHAP and Attention	Scalable AI for diagnostics and monitoring
Ennab & Mcheick (2024) [29]	Biomedical robotics	DL models	Visualization and Feature Attribution	Reviewed interpretability challenges and proposed safer AI strategies
Frasca et al. (2024) [20]	Multi‐domain	Various	SHAP and Grad‐CAM	Bibliometric review of 448 articles; emphasized trust and transparency
Holzinger et al. (2019) [2]	Oncology and genomics	Decision Trees	Causability and SHAP	Ethical framework for interpretable AI
Antoniadi et al. (2021) [30]	CDSS	Tabular ML models	Post hoc & Ante‐hoc	Reviewed XAI in CDSS; emphasized clinician usability gaps
Tjoa & Guan (2020) [21]	Multi‐domain	CNN, RNN, and Decision Trees	SHAP and LIME	Survey of medical XAI techniques and challenges
Adadi & Berrada (2018) [31]	General healthcare	Black‐box models	SHAP and LIME	Conceptual framework for XAI in healthcare
Ghassemi et al. (2021) [32]	ICU and EHR analysis	Deep Neural Networks	Feature Attribution	Critique of interpretability in clinical ML models
Topol (2019) [33]	Healthcare innovation	Various	Conceptual	Advocated for human‐centered AI in medicine
Nazir et al. (2024) [34]	Neurology and oncology	CNN and Transformer	SHAP and Grad‐CAM	PRISMA‐based review of 89 studies; identified gaps in data and workflow integration
Kabir et al. (2025) [35]	Infectious disease	XGBoost and LSTM	SHAP	AI predicts outbreak trends; climate and mobility factors emphasized
Yoo et al. (2023) [36]	Cardiology	CNN and LSTM	Grad‐CAM and SHAP	XAI improves interpretability of ECG‐based arrhythmia detection

Abbreviations: CDSS, clinical decision support system; CNN, convolutional neural network; DL, deep learning; Grad‐CAM, gradient‐weighted class activation mapping; LIME, local interpretable model‐agnostic explanations; LSTM, long short‐term memory network; ML, machine learning; RNN, recurrent neural network; SHAP, SHapley Additive exPlanations; XAI, explainable artificial intelligence; XGBoost, extreme gradient boosting.

The most commonly used explainability methods were SHAP (38 studies, 54%), LIME (21 studies, 30%), Grad‐CAM (16 studies, 23%), and attention mechanisms (14 studies, 20%). Some studies additionally incorporated rule‐based models, causability frameworks, and feature attribution techniques. Out of the 70 included studies, 12 explicitly addressed ethical and regulatory considerations, including bias, fairness, patient consent, and accountability, reflecting a growing attention to responsible and transparent AI in healthcare (see Tables 3 and 4). Representative studies were selected based on coverage of diverse healthcare domains, variety of AI model families, and inclusion of interpretable techniques, ensuring the examples capture the breadth of applications and illustrate trends, innovations, and gaps across the literature.

### 3.2 Applications of Explainable AI in Healthcare

Explainable AI methods were applied across multiple clinical contexts. In oncology and radiology, CNNs combined with Grad‐CAM or SHAP enabled the visualization of tumor regions and the identification of critical imaging features, enhancing interpretability for clinical decision‐making [34, 37]. In cardiology, RNNs and LSTMs with attention mechanisms improved the understanding of ECG signals and longitudinal patient data, supporting accurate risk assessment and personalized care [38, 39, 40, 41].

**Figure 2:**
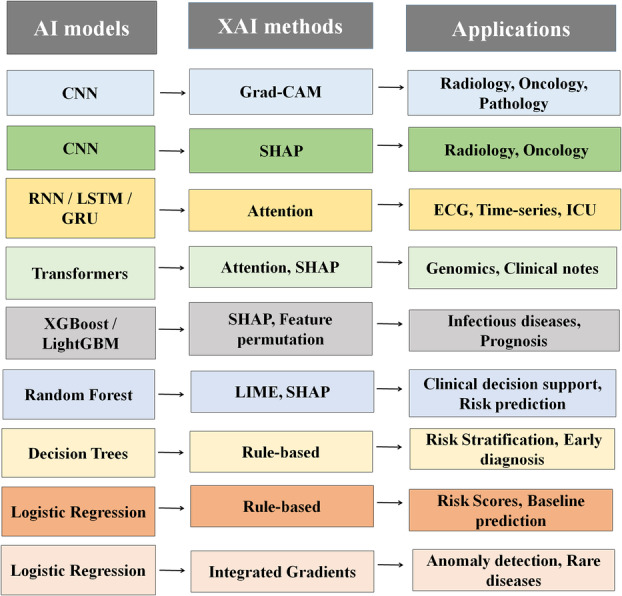
Mapping AI models to XAI techniques and healthcare applications. CNN, convolutional neural network; RNN, recurrent neural network; LSTM, long short‐term memory; GRU, gated recurrent unit; XGBoost, extreme gradient boosting; LightGBM, light gradient boosting machine; SHAP, SHapley Additive exPlanations; LIME, local interpretable model‐agnostic explanations; Grad‐CAM, gradient‐weighted class activation mapping; XAI, explainable artificial intelligence; ECG, electrocardiogram; ICU, intensive care unit.

In infectious disease prediction and prognosis, tree‐based models such as XGBoost and Random Forest were widely applied. SHAP and LIME highlighted the most influential clinical and demographic features [2, 22, 35, 42, 43]. For genomics and clinical note analysis, Transformer architectures facilitated the interpretation of sequential and text‐based data using attention mechanisms and SHAP, improving insights into complex molecular and patient‐level information [44, 45, 46]. Additionally, autoencoder‐based models employing Integrated Gradients were explored for anomaly detection and rare disease identification, demonstrating the potential of XAI to uncover hidden patterns that may otherwise go undetected [47, 48, 49]. The mapping of AI models to XAI techniques across healthcare domains is illustrated in Figure 2, highlighting the diversity of approaches and clinical applications.

### 3.3 Trends, Innovations, and Gaps

Several notable trends observed across the included studies. Hybrid models, which combine high‐performance AI with interpretable frameworks, are increasingly used to balance accuracy and transparency [50, 51, 52]. There is a growing emphasis on human‐centered and ethical design, with a focus on clinician‐friendly interpretability, bias mitigation, and fairness [53, 54, 55, 56]. Domain‐specific applications of XAI are becoming more prevalent, with methods tailored to imaging, genomics, time‐series data, and structured clinical datasets [57].

Despite these advances, several gaps persist. There is currently no standardized metric to measure interpretability, making cross‐study comparisons challenging. Many models have not been validated in real‐world clinical settings, limiting practical adoption. Some XAI outputs remain difficult for non‐technical healthcare professionals to interpret [29]. Furthermore, regulatory and ethical frameworks for the clinical deployment of XAI are still underdeveloped, limiting widespread adoption in healthcare practice.

### 3.4 Summary of Selected Studies

Table 4 provides an overview of representative studies on Explainable AI in healthcare. Notable examples include, Fahim et al. (2025) implemented CNN, LSTM, and XGBoost models with SHAP and attention mechanisms for scalable diagnostics and patient monitoring across multiple domains [22]. Ennab and Mcheick (2024) focused on biomedical robotics, reviewing interpretability challenges and proposing safer AI strategies [29]. Frasca et al. (2024) conducted a bibliometric review of 448 articles, emphasizing trust and transparency in multi‐domain applications [20]. Frasca et al. (2020) provided an ethical evaluation of explainability across clinical decision support systems [20], while Holzinger et al. (2019) proposed a framework for interpretable AI in oncology and genomics using decision trees with causability measures [2]. Other notable contributions include XAI applications in ICU and EHR analysis [32], genomic and neurological studies [34], infectious disease outbreak prediction [35], and arrhythmia detection in cardiology [36].

These studies collectively highlight the breadth of XAI applications, the diversity of models and techniques, and the emerging focus on ethical and human‐centered AI design in healthcare.

## 4 Discussion

This review highlights the pivotal role of Explainable Artificial Intelligence (XAI) in advancing transparency, accountability, and clinical trust in healthcare‐oriented AI systems. The integration of interpretability frameworks, such as SHAP, LIME, Grad‐CAM, and attention mechanisms has significantly improved the comprehension of complex machine learning models, effectively bridging the gap between computational sophistication and clinical applicability [12, 58, 59]. These findings reinforce the growing consensus that explainability is a critical requirement for the safe and responsible deployment of AI in healthcare settings.

The widespread adoption of deep learning architectures (CNNs, RNNs, LSTM networks, and Transformers) across diverse healthcare applications was evident in this review [6, 29, 37, 38, 60, 61]. When coupled with XAI approaches, these models have demonstrated strong potential in medical imaging, genomics, and electronic health record (EHR) analytics [17, 20, 29, 37, 38, 40, 62]. For example, CNN–Grad‐CAM combinations have enabled the visualization of tumor regions in diagnostic imaging, while SHAP and attention‐based explanations have clarified feature contributions in predictive modeling of disease progression and treatment outcomes [34, 63]. Such integrations not only improve model transparency but also support clinician understanding and decision‐making.

A notable evolution within this field is the rise of hybrid frameworks that fuse high‐performing AI models with interpretable components [64, 65]. These models aim to strike a balance between predictive precision and clinical interpretability, directly addressing the dual imperative of accuracy and transparency in healthcare AI. Such approaches align with emerging evidence suggesting that clinician confidence and adoption are closely linked to the clarity, consistency, and accessibility of model explanations [66, 67, 68]. As a result, hybrid and human‐centered XAI designs are gaining prominence as practical solutions for real‐world implementation.

Despite substantial progress, several challenges persist. The absence of standardized interpretability metrics hampers meaningful comparisons across XAI techniques [69]. Although many models demonstrate strong performance in controlled research environments, their clinical translation and validation remain limited [70]. Furthermore, the usability and cognitive burden of XAI outputs vary, with certain explanations proving technically demanding for non‐specialist healthcare users [71, 72, 73, 74]. Addressing these limitations necessitates user‐centered design, interdisciplinary collaboration, and targeted clinician training to ensure that explainability translates into practical clinical utility [75].

Ethical and regulatory considerations remain central to the responsible deployment of AI in healthcare [76]. The reviewed studies demonstrate a growing awareness of fairness, bias mitigation, and accountability, reflecting a shift toward responsible AI practices [77]. However, the development of comprehensive regulatory frameworks specifically addressing XAI remains nascent. Effective implementation will require coordinated action among clinicians, researchers, and regulators to establish transparent, enforceable, and ethically grounded standards for explainable AI systems.

Looking ahead, future research should prioritize the establishment of standardized interpretability benchmarks, conduct large‐scale clinical trials to evaluate model generalizability, and design intuitive XAI tools adaptable to varying levels of clinical expertise. Furthermore, the evolution of robust ethical and governance frameworks will be essential for the equitable, transparent, and sustainable integration of AI technologies into healthcare systems—ultimately fostering trust, patient safety, and improved health outcomes.

### 4.1 Limitations

This review has a few limitations. First, the literature search was limited to six major academic databases (Elsevier, Springer, Taylor & Francis, Semantic Scholar, ACM Digital Library, and IEEE Xplore), which may have left out significant research from other resources. Second, only peer‐reviewed publications published between 2017 and 2025 were considered, possibly excluding older fundamental work or extremely recent preprints. Third, the selected research are inherently heterogeneous, with variances in AI models, XAI approaches, healthcare areas, and assessment methods, making direct comparisons difficult. Finally, because of the methodological and reporting variability, this review did not perform a quantitative meta‐analysis, focusing instead on qualitative synthesis of study‐level findings.

## 5 Conclusion

This systematic review synthesized evidence from 70 peer‐reviewed studies published between 2017 and 2025 to examine how Explainable Artificial Intelligence (XAI) is being integrated into healthcare machine‐learning applications. The findings show that deep learning models (CNNs, recurrent architectures, and Transformer‐based systems) dominate current practice, with SHAP, LIME, Grad‐CAM, and attention mechanisms emerging as the most frequently used explanation techniques across clinical domains, such as oncology, cardiology, infectious diseases, genomics, and clinical decision support. Beyond mapping models to XAI methods and application areas, this review identifies clear cross‐study patterns and gaps. While explainability is increasingly incorporated to enhance transparency and clinician trust, only a minority of studies explicitly address ethical, regulatory, and fairness considerations, and real‐world clinical validation remains limited. In addition, the absence of standardized metrics for evaluating interpretability continues to hinder meaningful comparison across studies and methods.

Overall, this review contributes a structured synthesis of XAI applications in healthcare, highlighting both methodological trends and persistent limitations. Future research should prioritize standardized interpretability benchmarks, rigorous clinical validation, and the development of user‐centered explanation interfaces, alongside clearer ethical and regulatory guidance. Addressing these issues is essential for translating XAI‐enabled systems from experimental settings into transparent, trustworthy, and clinically actionable healthcare solutions.

## Author Contributions

**Md. Abu Bokkor Shiddik:** conceptualization, investigation, funding acquisition, writing – original draft, methodology, validation, visualization, writing – review and editing, software, formal analysis, project administration, data curation, supervision, resources.

## Funding

The author received no specific funding for this work.

## Ethics Statement

Not applicable, as this study is a systematic review of previously published research.

## Conflicts of Interest

The author declares no conflicts of interest.

## Transparency Statement

The lead author Md. Abu Bokkor Shiddik affirms that this manuscript is an honest, accurate, and transparent account of the study being reported; that no important aspects of the study have been omitted; and that any discrepancies from the study as planned (and, if relevant, registered) have been explained.

## Data Availability

Data sharing is not applicable to this article, as no new data were created or analyzed in this study.

## Appendix A Database‐Specific Search Strategy

### A.1

A systematic literature search was conducted across six electronic databases: Elsevier (ScienceDirect), SpringerLink, Taylor & Francis Online, Semantic Scholar, ACM Digital Library, and IEEE Xplore. Searches were limited to peer‐reviewed journal articles published between January 2017 and December 2025 and written in English.

The core Boolean search logic was adapted to the syntax of each database. An example of the search string used for IEEE Xplore is shown below:

(“explainable artificial intelligence” OR “explainable AI” OR XAI OR “interpretable machine learning” OR “model interpretability”)

AND

(“machine learning” OR “deep learning” OR “neural network*”)

AND

(healthcare OR medicine OR clinical OR diagnosis OR prognosis)

Equivalent keyword logic and Boolean operators were applied across the remaining databases with minor database‐specific modifications (e.g., field tags and wildcard usage). No geographic restrictions were applied at the search stage. Initial searches returned a large number of raw records across databases. Automated filters (publication type, year, and subject relevance) were applied to exclude non‐peer‐reviewed and out‐of‐scope records prior to screening. The final database searches were conducted between September 1, 2025, and October 15, 2025.

## Appendix B Screening and Study Selection Workflow

### B.1

All records retrieved from the six databases (Elsevier, Springer, Taylor & Francis, Semantic Scholar, ACM Digital Library, and IEEE Xplore) were exported and consolidated into a single reference library using Zotero reference management software. Table 2 reports the raw keyword search hits from each database. After consolidation and removal of duplicate records using Zotero's built‐in duplicate detection function followed by manual verification 302 unique records were identified for title and abstract screening, consistent with the PRISMA flow diagram (Figure 1).

To enhance efficiency and consistency, a Python‐based wrapper was used to extract bibliographic metadata (e.g., title, authors, year, and abstract) from the consolidated library. This preliminary automated extraction was followed by manual verification to ensure accuracy and completeness.

Title and abstract screening was conducted to assess relevance to healthcare‐focused ML/DL applications incorporating explainability or interpretability. Full‐text screening was then performed to confirm eligibility against predefined inclusion and exclusion criteria (see Table 1). Screening decisions were based on relevance to healthcare, use of ML or DL models, incorporation of XAI or interpretability techniques, and evidence of practical, clinical, or policy relevance. Screening and assessment were performed by a single reviewer, with re‐evaluation of ambiguous records to ensure consistency.

The final selection included 70 studies that met all criteria and were incorporated into the qualitative synthesis. The study selection workflow is summarized in the PRISMA flow diagram (Figure 1). This systematic review protocol was not registered in PROSPERO or any other review registry.

## Appendix C Extracted Study‐Level Variables

### C.1

For each included study, the following variables were systematically extracted and recorded:

i. Healthcare domain or application area.
ii. AI/ML model family (e.g., traditional ML, deep learning, and hybrid models).
iii. Explainability approach (e.g., SHAP, LIME, Grad‐CAM, attention mechanisms, rule‐based, or inherently interpretable models).
iv. Level of explainability (global and/or local).
v. Data type (e.g., imaging, clinical records, time‐series, and multimodal data).
vi. Validation strategy (e.g., internal validation, external datasets, and real‐world deployment).
vii. Reported outcomes and performance metrics.
viii. Ethical and regulatory considerations (e.g., bias, fairness, transparency, accountability, consent, and regulatory alignment).

These extracted variables formed the basis for the thematic synthesis and comparative evaluation presented in the Results and Discussion sections.

## Appendix D Data Availability and Transparency Statement

### D.1

No primary patient‐level data were generated or collected for this study. All analyses were based on data extracted from published peer‐reviewed articles. Extracted study‐level data are available from the corresponding author upon reasonable request.
